# Validation of the Korean Version of Impact of Event Scale—Revised (IES-R) in Korean Nurses during the COVID-19 Pandemic

**DOI:** 10.3390/ijerph182111311

**Published:** 2021-10-28

**Authors:** Young Suk Park, Kwang-Hi Park, Juna Lee

**Affiliations:** 1 Department of Nursing, Korea National Open University, Seoul 03087, Korea; anywayyoung@knou.ac.kr; 2 School of Nursing, Gacheon University, Incheon 21936, Korea; 3 Center for Human-Caring Nurse Leaders for the Future by Brain Korea 21 (BK21) Four Project, College of Nursing, Seoul National University, Seoul 03080, Korea

**Keywords:** COVID-19, epidemics, nurses, post-traumatic stress disorders, validation study, surveys and questionnaires

## Abstract

Nurses working amid the COVID-19 pandemic are at increased risk of developing post-traumatic stress disorder (PTSD). This study was conducted to verify the reliability and validity of the Korean version of Impact of Event Scale—Revised (IES-R), one of the most used tools for assessing trauma. Secondary data of 249 nurses who performed face-to-face nursing tasks during the COVID-19 pandemic, collected through an online survey, were analyzed by conducting a factor analysis of the K-IES-R and testing the internal consistency and concurrent validity with the Perceived Stress Scale (PSS), Generalized Anxiety Disorder Screener (GAD-7), and Dimensions of Anger Reactions-5 (DAR-5). The result of an exploratory factor analysis of the K-IES-R supported a three-factor structure of intrusion, avoidance, and sleep disturbance, with CMIN/DF = 2.98, RMSEA = 0.09, SRMR = 0.03, CFI = 0.93, and TLI = 0.90. The Cronbach’s alpha of each subscale was 0.88–0.94. The total K-IES-R score and each factor’s value showed a significant correlation (moderate or higher) with the PSS, GAD-7, and DAR-5. The K-IES-R was verified as a useful tool for assessing post-traumatic stress symptoms in nurses who directly perform nursing tasks in crises such as COVID-19. This study suggests the tool be used for early assessment of post-traumatic stress symptoms in nurses and providing appropriate interventions.

## 1. Introduction

According to the World Health Organization, Coronavirus disease (COVID-19) started in December 2019 and as of 16 May 2021, there have been more than 160 million cumulative confirmed positive cases and 3.3 million cumulative deaths worldwide [1]. Nationwide health problems caused by unstoppable COVID-19 transmission and infections pose a psychological burden on healthcare personnel (HCP), including nurses [2]. According to a systematic literature review conducted based on previous studies, the incidence of depression, anxiety, and stress among HCP engaging in COVID-19 treatment amounted to approximately 24.3, 25.8, and 45.0%, respectively [2]. It has also been confirmed that socio-demographic characteristics and work experience, a rapidly increasing number of critically ill patients and high mortality rates, the burden of decision-making and uncertainty, and the demand for up-to-date information can cause post-traumatic stress disorders (PTSD) in HCP, making early assessment imperative [3]. In particular, there are studies [4,5] that report prolonged and repeated anxiety, depression, fear, anger, guilt, tough decisions, and ethical conflicts experienced while performing COVID-19-related tasks in situations that are short of human resources and protective equipment can cause PTSD in nurses.

The Impact of Event Scale—Revised (IES-R) is one of the most commonly used tools to assess the health of people who have experienced trauma [6]. It was first developed by Horowitz et al. [7] in the form of a 15-item self-report questionnaire and was revised by Weiss et al. [8], who added six items related to hyperarousal symptoms [9]. Later, the IES-R has been translated into various language including Chinese [10], Swedish [11], French [12], and Korean [6,9], and applied to groups of various characteristics such as earthquake victims [13], burn patients [11], and veterans [14], with its statistical validity being evaluated. Recently, there were attempts to confirm its applicability to situations of novel infectious disease transmission such as COVID-19 [15]. However, there have not been any reliability and validity tests to use it for early assessment of mental health problems and PTSD in nurses coping with the COVID-19 pandemic situation.

There have been many studies exploring the latent structure of the IES-R and proposing diverse competing models for it, such as the five-factor-numbing model, the five-factor-dysphoria model, the DSM-IV-three factor model, and the DSM-IV model with a separate sleep problems model [16]. Testing the reliability and validity of the models, previous studies agreed that the factor solutions for the IES-R were affected by cultural backgrounds [16].

If the reliability, validity, and underlying dimensional structure of the IES-R are verified by applying it to HCP coping with COVID-19 transmission, especially to nurses who have relatively long contact, it will contribute to the early detection and appropriate management of PTSD in nurses who perform nursing tasks among the spread of novel infectious diseases such as SARS-CoV (severe acute respiratory syndrome coronavirus) and MERS-CoV (Middle East respiratory syndrome coronavirus), that are in the same viral family, in the future. Therefore, this study was designed to examine the psychometric properties, reliability, and convergent validity of the IES-R in Korean nurses during the COVID-19 pandemic.

## 2. Materials and Methods

The study received approval from the university’s institutional review board before analyzing the data to test the reliability and validity of the IES-R. This study was based on secondary data collected using convenience sampling and an online survey from January 2020 to March 2021. Recruitment notices were posted in an online community where more than 84,000 Korean nurses subscribed and interacted with each other. It was distributed purposely to related nurses using social media services in consideration of the COVID-19 outbreak areas across the country and the institution type. Nurses who saw the recruitment notice and voluntarily wanted to participate in the study could access the research platform through the linked address.

### 2.1. Participants

The inclusion criteria of participants was as follows: nurses who performed nursing tasks for at least one month in a tertiary general hospital, general hospital, long-term care hospital, public health center, or a screening clinic during the COVID-19 pandemic, coming face to face with confirmed or suspected COVID-19 patients. The study excluded nurses who were performing COVID-19-related tasks not because they were assigned to them but because they volunteered. It is known that the number of samples required to statistically test the reliability and validity of a tool is at least five times the number of its items [17]. Since the IES-R has 22 items, the number of samples required for the study was 100, which the study satisfied.

### 2.2. Variables and Measures

#### 2.2.1. Impact of Event Scale—Revised, IES-R

The IES-R was developed by Horowitz et al. [7] and revised by Weiss et al. [8] into a 22-item self-report scale including 8 items for intrusion, 8 items for avoidance, and 6 items for hyperarousal, each being rated on a 5-point (0–4) scale. Sometimes, however, the items have been classified differently depending on the exploratory or confirmatory factor analysis carried out by various studies [6,9,14,16]. Higher scores are interpreted as having more severe post-traumatic stress.

Eun et al. [6] and Lim et al. [9] had translated the IES-R into Korean, and Park et al. [18] revised the adapted scales based on consultations with psychiatric specialists to improve the content validity and face validity of the tools. This study applied the tool developed by Park et al. [18] to nurses who performed COVID-19-related tasks and conducted confirmatory factor analysis referring to the results of Weiss et al. [8], Eun et al. [6], and Lim et al. [9], as well as additional exploratory factor analysis. This study used 18 points as the cutoff score for partial PTSD, which Eun et al. [6] had reported.

#### 2.2.2. Perceived Stress Scale, PSS

It is known that PTSD or the values measured using the IES-R have significant correlation with the level of acute stress or subjective stress [19,20]. Therefore, this study, to evaluate the construct validity of the IES-R, looked at whether it had a statistical correlation with stress measured by the Perceived Stress Scale (PSS) [21,22]. The PSS is a self-report 5-point (0–4) scale consisting of 10 items, with a minimum score of 0 and a maximum of 40. Higher scores indicated a higher level of perceived stress [22]. The tool was not developed for diagnostic purposes and is known to have no specific cutoff scores suggested [22].

#### 2.2.3. Generalized Anxiety Disorder Screener, GAD-7

Based on previous studies that reported PTSD which developed in the context of COVID-19 pandemic was associated with anxiety [6,9], this study, to evaluate the construct validity of the IES-R, looked at its statistical correlation with anxiety measured by the Generalized Anxiety Disorder Screener (GAD-7) [23]. In Korea, the GAD-7 was adapted and standardized by Seo et al. [24] by applying it to epilepsy patients. It is a self-report 4-point scale with 7 items and higher scores indicate greater anxiety [24]. The strict cutoff score is reported to be 15 points and the high sensitivity cutoff score to be 10 points [23].

#### 2.2.4. Dimensions of Anger Reactions-5, DAR-5

This study used the Dimensions of Anger Reactions-5 (DAR-5) which was developed by Forbes et al. [25] and adapted by Park et al. [18] to evaluate the construct validity of the IES-R by looking into the statistical correlation between the IES-R results and anger. The DAR-5 is a self-report 4-point (1–5) scale with 4 items, with a total score ranging from 5 to 20. A score of 12 or higher is interpreted as an angry reaction in PTSD [18].

#### 2.2.5. General Characteristics of Participants

The study asked the participants for information about their socio-demographic (gender, age, marital status, education) and job characteristics (position, working period, COVID-19-related task period, institution) and used the data for analysis.

### 2.3. Statistical Procedures

Statistical analysis was performed with a 95% confidence level, using IBM SPSS statistics version 25.0 and Mplus 8.4 (Muthen and Muthen, 2017). General characteristics were analyzed using descriptive statistics such as frequency, percentage, mean, and standard deviation.

Cronbach’s alpha was calculated to confirm the internal consistency of the tool. Then, the corrected item-total correlation coefficient was calculated to see whether there were any items with a value lower than the reliability coefficient or an item-total correlation coefficient less than 0.3. The Kaiser-Meyer-Olkin (KMO) test and Bartlett’s test of sphericity were conducted to test the goodness of fit of the data, and factor analysis was performed using eigenvalues, a scree plot, and the maximum likelihood method, applying an oblique goemin rotation method. The validity of the tool, referring to the guide proposed by Steiger [26], Kline [27], and Jr., et al. [28] was evaluated as follows: CMIN/DF (≤3), Root Mean Square Error of Approximation (RMSEA, ≤0.10), Comparative Fit Index (CFI, ≥0.90), Tucker–Lewis Index (TLI, ≥0.90), and Standardized Root Mean Square Residual (SRMR, ≤0.08). In exploratory factor analysis, the Acaike Information Criterion (AIC) and Bayesian Information Criterion (BIC) were confirmed to evaluate trade-off between the goodness of fit indices and the degree of freedom and to select among competing models [27]. Then, the study examined whether the Pearson correlation coefficients between the values measured by the IES-R and those measured by the PSS, GAD-7, and DAR-4 were ≥0.45 (*p* < 0.05), which considered substantial for convergent validity [29].

### 2.4. Ethical Consideration

The study used secondary data and was conducted after receiving approval from the institutional review board (ABN01-202106-21-10) of the institution to which the researcher belongs. The secondary data used in the study did not contain any personally identifiable information.

## 3. Results

### 3.1. Characteristics of Participants of Internet Survey

A total of 268 nurses responded to the survey. Among them, data of 249 participants were used for statistical analysis after excluding duplicates or incomplete responses. Of the 249 participants, the mean age was 31.42 (SD = 8.47) years and most of them were female (n = 225, 90.4%). Among participants, 71.1% were single (n = 177) and 69.1% had a bachelor’s degree (n = 172).

On average, the participants spent time on COVID-19-related tasks for 6.47 (SD = 3.77) months. The average career length as a nurse was 7.32 (SD = 7.60) years, with the shortest being 2 months and the longest being 34 years. Two hundred twenty-eight participants (91.6%) were staff or charge nurses and sixteen (6.4%) responded they worked for a public institution or worked on a fixed-term basis. Ninety-three nurses (31.2%) performed COVID-19-related tasks in tertiary general hospitals, one hundred and ten (36.9%) in general hospitals, thirty-eight (12.8%) in public health centers, and seven (2.3%) in long-term care hospitals. Thirty-seven nurses (12.4%) had experience working at COVID-19 screening clinics and thirteen nurses (4.4%) had experience working at other institutions such as community treatment centers for people with mild COVID-19 symptoms, 119 emergency services, etc.

The average score measured by the IES-R was 17.66 (SD = 15.98). When applying the high sensitivity cutoff score suggested by Eun et al. [6], which is 18 points, 100 participants (40.2%) had PTSD. The average perceived stress measured by PSS was 21.39 (SD = 6.08) and anxiety measured by GAD-7 was 6.08 (SD = 5.44). When applying the high sensitivity cutoff score, which is 10 points [18], 54 participants (21.7%) had anxiety disorders. The average anger measured by the DAR-5 was 9.56 (SD = 4.37) and 72 participants (28.9%) had a score of ≥12, as shown in Table 1.

### 3.2. Confirmatory Factor Analysis

For the IES-R, exploratory factor analysis has already been conducted by Weiss et al. [8], Lim et al. [9], and Eun et al. [6], as shown in Table 2. This study, to test the construct validity of the IES-R, conducted confirmatory factor analysis based on the three previous studies. To do so, the KMO test for sampling adequacy and Bartlett’s test of sphericity were first performed to confirm the goodness of fit of the factor model (KMO = 0.950, χ^2^ = 4463.74, *p* < 0.001).

As a result, the fit of the factor model suggested by Weiss et al. [8] was CMIN/DF = 4.09, RMSEA = 0.11, CFI = 0.86, TLI = 0.84, SRMR = 0.06. The fit of the factor model suggested by Lim et al. [9] was CMIN/DF = 3.99, RMSEA = 0.11, SRMR = 0.06, CFI = 0.86, TLI = 0.90, and the result based on Eun et al. [6] was CMIN/DF = 4.04, RMSEA = 0.11, SRMR = 0.06, CFI = 0.86, TLI = 0.84. There was no significant difference among the three studies and the statistical validity was evaluated low.

### 3.3. Exploratory Factor Analysis

Cronbach’s alpha was calculated to check the internal consistency of the tool, which was found to be at an acceptable level, α = 0.94. Then, the study examined whether there were any cases in which the item reliability coefficient was lower than the tool reliability coefficient or the corrected item-total correlation coefficient was less than 0.3, which turned out to be none.

**Figure 1:**
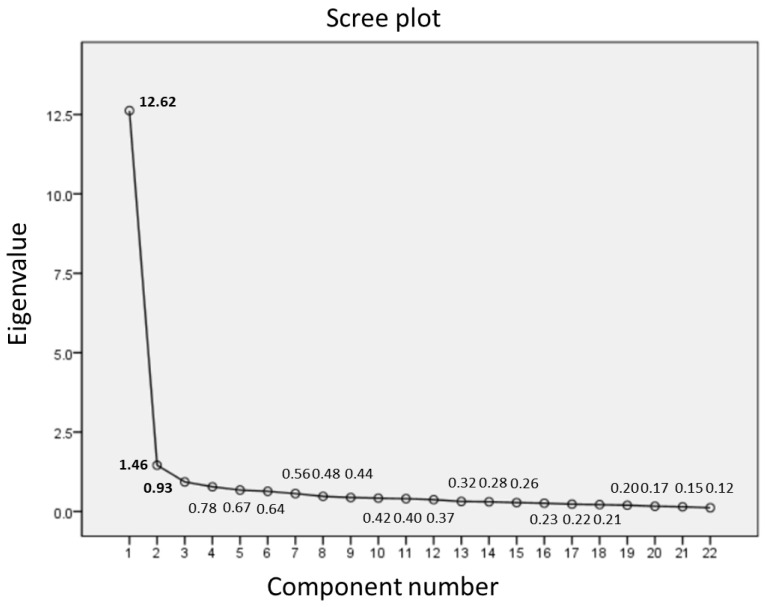
Scree plot of the eigenvalues.

Looking at the Scree test result and eigenvalues, the data were classified into two factors (Eigenvalue = 12.62; 1.46) as shown in Figure 1. However, since the third factor’s eigenvalue was on the borderline close to 1 (Eigenvalue = 0.93), the study also conducted an exploratory factor analysis based on a three-factor structure.

The fit of the two-factor model was CMIN = 654.30, df = 188, CMIN/DF = 3.48, RMSEA = 0.10, SRMR = 0.04, CFI = 0.89, TLI = 0.87, AIC = 11175.01, BIC = 11481.03, and the fit of the three-factor model was CMIN = 499.83, df = 168, CMIN/DF = 2.98, RMSEA = 0.09, SRMR = 0.03, CFI = 0.93, TLI = 0.90, AIC = 11060.54, BIC = 11436.91. Additionally, it was difficult to identify each structure by the result of a two-factor analysis (Appendix A). Therefore, the study decided to use three factors. The factor loading and internal consistency of the items are shown in Table 3.

### 3.4. Convergent Validity

The total and each factor’s score measured by the IES-R showed a significant (moderate or higher) correlation with subjective stress (r = 0.48–0.61, *p* < 0.001), anxiety (r = 0.51–0.72, *p* < 0.001), and anger (r = 0.48–0.67, *p* < 0.001) measured by the PSS, GAD-7, and DAR-5, respectively (Table 4).

## 4. Discussion

The study evaluated the reliability, validity, and discovered the latent structure of the IES-R as part of an approach to come up with an appropriate response plan by early assessment of PTSD in nurses caused by nationwide health problems during the COVID-19 pandemic. The findings identified a three-factor model with distinct factors of intrusion, avoidance, and sleep disturbance as the best fitting model. In the study, the IES-R showed a higher internal consistency (Cronbach’s α = 0.96) than the report from the previous studies performed in Korea (Cronbach’s α = 0.83–0.93) [6,9]. In the correlation analysis to test its construct validity, it was evaluated as a valid tool, showing moderate or higher correlations with stress, anxiety, and anger [6,9,19,20].

In the process of determining the number of factors based on the Scree test result and eigenvalues, our results suggested that a two-factor solution was statistically acceptable for the IES-R. However, the result of a two-factor analysis did not exhibit the structures where each factor was distinguished, although the target outcome of exploratory factor analysis is a solution that provides a simple structure, where each factor has as much variance as possible in non-overlapping sets of indicators [27]. In consideration of the theoretical validity proposed by previous studies, our study conducted a factor analysis based on three factors and found that the CFI and TLI reached scores indicating the more acceptable model fit, ≥0.90 [28]. The three-factor model was consistent with the proposed theoretical structure of the scale, with an improved statistical model fit.

On the other hand, the factor analysis result for the IES-R showed some differences from those of previous studies, which has brought about active discussion about the reason, with the argument calling for more studies to accumulate evidence gaining strength [12]. In this study, the hyperarousal factor, which was added at the time the IES-R was developed [8], was not statistically confirmed. However, most of the items belonging to hyperarousal coincided with the result of a previous study [8] where they loaded on intrusion or avoidance, while two items related to sleep was classified into a new factor, sleep disturbance.

Some researchers mentioned that more studies were required to find the standard solution on this point [9]. This study agrees with it to a degree but values such differences in the results simultaneously. For instance, sleep disorders or sleep disturbance have been identified as one of the factors of the IES-R more frequently in Asian cultures compared to other cultural backgrounds [6,9,12]. Grassi et al. [16] also found sleeping disorders as one of the factors of the IES-R if the circumstances where the refugee was living were uncomfortable and might have enhanced their sleep problem. This study focused on nurses in the COVID-19 pandemic situation and discovered sleeping disturbance as one factor. It was reported that trauma-related sleep disorders among healthcare workers, including nurses, had been quickly found during the COVID-19 pandemic [30]. Thus, the researchers need to pay attention to the differences and try to understand the characteristics of traumatic symptoms depending on each person because they might reflect contextual variables and cultural backgrounds [16]. There is not yet a standard structure for the IES-R which all researchers agree on. Considering occupational and cultural situations, we can use the factors identified in this study as a subscale with closer attention, and it required additional repetitive studies to confirm this.

Lim et al. [9] applied the IES-R not only to patients diagnosed with psychiatric disorders but to the healthy control group. On the other hand, Eun et al. [6] applied the IES-R to college students and neuropsychiatric and orthopedic patients classified into criteria A (exposed to the stressor) based on the DSM-IV, who are homogeneous participants. Lim et al. [9] and Eun et al. [6]’s studies which this study adopted for the confirmatory factor analysis differed in the results in some aspects. Further studies are needed to elucidate the reason, but it is supposed that the homogeneity of participants might have affected the factor analysis of the IES-R. Therefore, researchers need to be careful and consider the characteristics and heterogeneity of the samples when applying and utilizing the IES-R.

Cases where items classified into hyperarousal by Weiss et al. [8] but loaded on other factors, as in this study, can be also seen in other studies [6,9,12,14]. One of them raised a possibility, as one of the reasons, that expressions such as “I felt” or “I had a feeling” in some hyperarousal items were perceived as symptoms or signs by the respondent, influencing the result of the confirmatory factor analysis. Another study argued that since there are cases where the hyperarousal factor is divided into numbness and sleep disturbance, it is necessary to reexamine the properties of the items [14]. In fact, there are a number of studies in which the factors, numbness and sleep disturbance, were confirmed [6,9,14]. This partially coincides with the present study where some items related to sleep were classified into a new independent factor [16], which is thought as a useful aspect that helps nurses to recognize and screen reactions to traumatic events more easily and clearly. In addition, Eun et al. [6] reported that the phenomenon where numbness and sleep disturbance are classified as independent factors is a tendency that is unique to Koreans, which calls for consideration of the socio-cultural background of Korean nurses in future research.

Item 5 (I avoided letting myself get upset when I thought about it or was reminded of it) was classified into avoidance by Weiss et al. [8], but loaded on intrusion in this study, which may have been affected by the expression “reminded”. This is the same as the study by Brunet et al. [12], which, we speculate, is because even though the former part describing “the behavior to avoid the problem” may be related to avoidance, the latter part of the sentence describing “the behavior of thinking or being reminded of the event” can be related to intrusion. Meanwhile, the translation process needs to be reexamined; it retained the rationale of the development stage but loaded on avoidance [8]. This was the same as the study by Brunet et al. [12] and, as mentioned earlier, the expression such as “acting or feeling” may have been perceived as symptoms or signs, influencing the result. However, since it was classified into numbness/dissociation in Lim et al. [9]’s study and into hyperarousal in Eun et al. [6]’s study, it is necessary to reexamine the content validity and translation process to determine whether it was faithful to the developer’s theoretical rationale and intent.

Item 19 (reminders of it caused me to have physical reactions, such as sweating, trouble breathing, nausea, or a pounding heart) loaded on avoidance in this study and the average response to Item 19 was 0.31 (SD = 0.63), the lowest among all IES-R items. This brings a previous study [31] to consideration, which conducted Latent Profile Analysis (LPA) based on IES-R responses to classify the severity of PTSD symptoms and reported that somatic symptoms tended to appear in the group judged to have severe PTSD symptoms. In other words, noticeable physical reactions such as sweating, difficulty breathing, nausea, or palpitations were low in the present study, whereas sleep disturbance, having Items 2 and 5 as subfactors, was high, with an average response of 1.26 (SD = 1.15) and 1.22 (SD = 1.23), respectively. In addition, Item 4 (I felt irritable and angry) showed the highest average response of 1.45 (SD = 1.19). Since these may be reactions unique to Korean nurses during the COVID-19 pandemic, it requires repeated studies for reconfirmation.

In a validity test of the tool adapted by Eun et al. [6], the strict cutoff score was reported to be 25 points and the cutoff score for partial PTSD to be 18 points [6]. Lim et al. [9] tested the reliability and validity of their adapted tool. In the ROC curve, the AUC was 0.91 and the cutoff score was 22 points [9]. Since the primary purpose of using the tool for nurses was early and quick detection of PTSD, we considered 18 points as a more sensible cutoff score for partial PTSD. The study used secondary data; it has a limitation that it was not able to use PTSD clinical screening tools based on interviews to confirm its concurrent validity. It is necessary to consider aspects that could not be interviewed due to the rapid and severe COVID-19 pandemic situation of the medical field and the nurses. Further studies should be conducted to reconfirm the accuracy and cutoff scores of clinical diagnoses of PTSD in nurses. This, along with the present study, will ultimately contribute to providing appropriate clinical interventions for nurses suffering from PTSD in epidemic situations.

The study attempted to recruit nurses working in diverse COVID-19-related fields and regions but adopted an online survey using convenience sampling to collect the data. If people were willing to participate in the survey but lacked the opportunities to access recruit notices, it could limit the participant pool. Additionally, nurses with more PTSD symptoms might have been likely to participate in the survey. However, in the sense that this study was the first trial that tested the IES-R to nurses during the COVID-19 pandemic in Korea, it would contribute to additional studies for using and evaluating this scale.

We recommend that the IES-R and factors of this study should apply to research and evidence building for early assessment of the mental health problems and needs of nurses responding to the COVID-19 pandemic, and in particular, for adequate policies and management of nurses’ PTSD in situations of infectious disease transmission.

## 5. Conclusions

The Korean version of IES-R (K-IES-R) showed high internal consistency of reliability in assessing PTSD in nurses who are providing direct care during the COVID-19 pandemic. It was also at an acceptable validity level and was found, from the factor analysis, to be a useful tool consisting of three factors: intrusion, avoidance, and sleep disturbance. We suggest follow-up studies on the recommended K-IES-R of this study be conducted and actively used to assess PTSD, stress, anxiety, and anger related to COVID-19 in nurses and to provide interventions.

## Figures and Tables

**Table 1:** General Characteristics of the Participants (N = 249).

Variables	Categories	Frequency (%)	Mean (SD)
**Socio-demographic characteristics**			
Gender	Male	24 (9.6)	
	Female	225 (90.4)	
Age (years)			31.42 (8.47)
	20–29	145 (58.2)	
	30–39	65 (26.2)	
	40≤	39 (15.6)	
Marital Status	Single	177 (71.1)	
	Married	72 (28.9)	
Highest education level	Associate degree	51 (20.5)	
	Bachelor’s degree	172 (69.1)	
	Postgraduate student	11 (4.4)	
	Master’s or doctoral degree	15 (6.0)	
**Job characteristics**			
COVID-19 -related task period (months)			6.47 (3.77)
	<6	88 (35.4)	
	6≤, <12	84 (33.7)	
	12≤	77 (30.9)	
Years of Experience			7.32 (7.60)
	<1	21 (8.4)	
	1≤, <3	54 (21.7)	
	3≤, <5	41 (16.4)	
	5≤, <10	37 (14.9)	
	10 ≤	96 (38.6)	
Position	Staff	228 (91.6)	
	Manager	5 (2.0)	
	Others	16 (6.4)	
Institution ^1^	Tertiary general hospital	93 (31.2)	
	General hospital	110 (36.9)	
	Public health center	38 (12.8)	
	Temporary screening clinic	37 (12.4)	
	Long-term care hospital	7 (2.3)	
	Others	13 (4.4)	
**Mental health characteristics**			
IES-R			17.66 (15.98)
	<18	149 (59.8)	
	18≤	100 (40.2)	
PSS			21.39 (6.08)
GAD-7			6.08 (5.44)
	<10	195 (78.3)	
	10≤	54 (21.7)	
DAR-5			9.56 (4.37)
	<12	177 (71.1)	
	12≤	72 (28.9)	

SD = standard deviation; ^1^ multiple responses were allowed.

**Table 2:** Previous studies which conducted exploratory factor analysis for IES-R.

Items	Weiss et al. [8]	Lim et al. [9]	Eun et al. [6]
1. Any reminder brought back feelings about it.	I	I	I
2. I had trouble staying asleep.	I	H	S/N
3. Other things kept making me think about it.	I	I	I
4. I felt irritable and angry.	H	I	H
5. I avoided letting myself get upset when I thought about it or was reminded of it.	A	A	A
6. I thought about it when I didn’t mean to.	I	I	I
7. I felt as if it hadn’t happened or wasn’t real.	A	N/D	S/N
8. I stayed away from reminders about it.	A	I	A
9. Pictures about it popped into my mind.	I	I	I
10. I was jumpy and easily startled.	H	H	H
11. I tried not to think about it.	A	A	A
12. I was aware that I still had a lot of feelings about it, but I didn’t deal with them.	A	A	A
13. My feelings about it were kind of numb.	A	N/D	S/N
14. I found myself acting or feeling like I was back at that time.	I	N/D	H
15. I had trouble falling asleep.	H	H	S/N
16. I had waves of strong feelings about it.	I	I	I
17. I tried to remove it from my memory.	A	A	A
18. I had trouble concentrating.	H	H	H
19. Reminders of it caused me to have physical reactions, such as sweating, trouble breathing, nausea, or a pounding heart.	H	I	H
20. I had dreams about it.	I	H	S/N
21. I felt watchful and on guard.	H	H	H
22. I tried not to talk about it.	A	A	A

IES-R = Impact of Event Scale—Revised; I = intrusion; H = hyperarousal; A = avoidance; N/D = numbness/dissociation; S/N = sleep and numbness.

**Table 3:** The modified model based on exploratory factor analysis.

Items	Mean (SD)	Correlation with Total	α If Item Deleted	Factor 1	Factor 2	Factor 3
**Intrusion (Cronbach’s alpha = 0.933)**						
1. Any reminder brought back feelings about it.	0.98 (0.90)	0.700	0.961	0.76 *	0.00	−0.02
3. Other things kept making me think about it.	0.87 (0.91)	0.756	0.961	0.74 *	0.01	0.09
4. I felt irritable and angry.	1.45 (1.19)	0.691	0.962	0.70 *	−0.13	0.26 *
5. I avoided letting myself get upset when I thought about it or was reminded of it.	0.86 (1.03)	0.803	0.960	0.87 *	0.03	−0.06
6. I thought about it when I didn’t mean to.	0.93 (1.04)	0.813	0.960	0.87 *	0.03	−0.06
9. Pictures about it popped into my mind.	0.92 (1.01)	0.791	0.960	0.88 *	0.02	−0.06
16. I had waves of strong feelings about it.	0.73 (0.94)	0.813	0.960	0.69 *	0.16	0.01
20. I had dreams about it.	0.73 (0.97)	0.733	0.961	0.43 *	0.30	0.09
21. I felt watchful and on guard.	0.85 (1.01)	0.683	0.961	0.34 *	0.29	0.16
**Avoidance (Cronbach’s alpha = 0.937)**						
7. I felt as if it hadn’t happened or wasn’t real.	0.59 (0.89)	0.673	0.961	0.07	0.68 *	−0.01
8. I stayed away from reminders about it.	0.70 (0.99)	0.795	0.960	0.32 *	0.55 *	0.00
10. I was jumpy and easily startled.	0.66 (0.95)	0.722	0.961	0.31 *	0.36 *	0.17 *
11. I tried not to think about it.	0.81 (0.99)	0.801	0.960	0.40 *	0.47 *	0.01
12. I was aware that I still had a lot of feelings about it, but I didn’t deal with them.	0.73 (0.97)	0.758	0.961	0.33 *	0.37 *	0.19 *
13. My feelings about it were kind of numb.	0.62 (0.92)	0.678	0.961	−0.25	0.87 *	0.23
14. I found myself acting or feeling like I was back at that time.	0.55 (0.88)	0.722	0.961	0.05	0.78 *	−0.05
17. I tried to remove it from my memory.	0.49 (0.75)	0.717	0.961	0.12	0.72 *	−0.05
18. I had trouble concentrating.	0.85 (0.96)	0.788	0.960	0.02	0.68 *	0.26 *
19. Reminders of it caused me to have physical reactions, such as sweating, trouble breathing, nausea, or a pounding heart.	0.31 (0.63)	0.559	0.963	−0.02	0.71 *	-0.11
22. I tried not to talk about it.	0.55 (0.85)	0.741	0.961	0.13	0.71 *	-0.02
**Sleep disturbance (Cronbach’s alpha = 0.876)**						
2. I had trouble staying asleep.	1.26 (1.15)	0.580	0.963	0.15	0.01	0.80 *
15. I had trouble falling asleep.	1.22 (1.23)	0.664	0.962	0.00	0.27 *	0.75 *

* significant at 5% level.

**Table 4:** Correlation of the IES-R with other variables (N = 249).

Factors	No. of Items	r (*p*)		
		PSS	GAD-7	DAR-5
Total score	22	0.58 (<0.001)	0.72 (<0.001)	0.67 (<0.001)
Intrusion	9	0.61 (<0.001)	0.72 (<0.001)	0.67 (<0.001)
Avoidance	11	0.49 (<0.001)	0.67 (<0.001)	0.61 (<0.001)
Sleep disturbance	2	0.48 (<0.001)	0.51 (<0.001)	0.48 (<0.001)

PSS = perceived stress score; GAD-7 = Generalized Anxiety Disorder screener; DAR-5 = Dimensions of Anger Reactions-5.

## Data Availability

Not applicable.

## Appendix A

**Table A1:** The result of exploratory factor analysis based on a two-factor structure.

Items	Factor 1	Factor 2
**Factor 1. Intrusion and avoidance**		
14. I found myself acting or feeling like I was back at that time.	0.873 *	−0.187 *
22. I tried not to talk about it.	0.868 *	0.137 *
17. I tried to remove it from my memory.	0.867 *	0.169 *
8. I stayed away from reminders about it.	0.861 *	−0.049
11. I tried not to think about it.	0.840 *	−0.013
16. I had waves of strong feelings about it.	0.828 *	0.011
6. I thought about it when I didn’t mean to.	0.787 *	0.068
5. I avoided letting myself get upset when I thought about it or was reminded of it.	0.785 *	0.067
7. I felt as if it hadn’t happened or wasn’t real.	0.781 *	−0.125
9. Pictures about it popped into my mind.	0.761 *	0.080
19. Reminders of it caused me to have physical reactions, such as sweating, trouble breathing, nausea, or a pounding heart.	0.748 *	−0.245 *
18. I had trouble concentrating.	0.699 *	0.154 *
20. I had dreams about it.	0.682 *	0.097
13. My feelings about it were kind of numb.	0.660 *	0.048
1. Any reminder brought back feelings about it.	0.654 *	0.092
12. I was aware that I still had a lot of feelings about it, but I didn’t deal with them.	0.642 *	0.197 *
3. Other things kept making me think about it.	0.634 *	0.205 *
10. I was jumpy and easily startled.	0.618 *	0.163 *
21. I felt watchful and on guard.	0.583 *	0.167 *
4. I felt irritable and angry.	0.430 *	0.406 *
**Factor 2. Sleep disturbance**		
2. I had trouble staying asleep.	0.006	0.891 *
15. I had trouble falling asleep.	0.191 *	0.732 *

* significant at 5% level.
